# High-Resolution Computed Tomography in Middle Ear Cholesteatoma: How Much Do We Need It?

**DOI:** 10.3390/medicina59101712

**Published:** 2023-09-25

**Authors:** Eugen Horatiu Stefanescu, Nicolae Constantin Balica, Sorin Bogdan Motoi, Laura Grigorita, Madalina Georgescu, Gheorghe Iovanescu

**Affiliations:** 1 Department of Otolaryngology, Victor Babeş University of Medicine and Pharmacy, 300041 Timişoara, Romania; stefanescu@umft.ro (E.H.S.); balica@umft.ro (N.C.B.); giovanescu@umft.ro (G.I.); 2 Department of Radiology and Medical Imaging, Victor Babeş University of Medicine and Pharmacy, 300041 Timişoara, Romania; motoi.sorin@umft.ro; 3 Department of Anatomy and Embriology, Victor Babeş University of Medicine and Pharmacy, 300041 Timişoara, Romania; grigorita.laura@umft.ro; 4 Discipline of General Surgery and Qualified Care in Surgical Specialties, Carol Davila University of Medicine and Pharmacy, 050474 Bucureşti, Romania

**Keywords:** high-resolution computed tomography, cholesteatoma surgery, very good correlation

## Abstract

*Background and Objectives*: The diagnosis of cholesteatoma is usually clinic, and the only efficient treatment is surgical. High-resolution computed tomography (HRCT) is not considered absolutely necessary for the management of an uncomplicated cholesteatoma, but unsuspected situations from a clinical point of view can be discovered using the scans, warning the surgeon. Our objective is to compare HRCT scan information with intraoperative findings in patients with cholesteatoma and analyze the usefulness of a preoperative HRCT scan from a surgical point of view. *Materials and Methods*: This is a prospective descriptive study conducted in the Department of Otolaryngology, Victor Babes University of Medicine and Pharmacy Timisoara, Romania, from May 2021 to April 2022. It was carried out on 46 patients with a clinical diagnosis of cholesteatoma who were consequently operated on in our department. All patients received full clinical and audiological examinations. In all cases, an HRCT scan was performed preoperatively as a mandatory investigation. Preoperative HRCT scans were analyzed, and their findings were compared to the intraoperative notes. The two sets of observations were analyzed using standard statistical methods. *Results*: Extensive cholesteatoma was the most common type of disease, involving 46% of the patients, followed by pars flaccida cholesteatoma (35%) and pars tensa cholesteatoma (19%). Eroded scutum was the most frequent lesion involving 70% of the patients, followed by incus erosion (67%). Comparison of the HRCT and intraoperative findings revealed a very good correlation for tegmen tympani erosion, sigmoid plate erosion, scutum and malleus erosion, and a moderate-to-good correlation for lateral semicircular canal erosion, incus and stapes erosion, and fallopian canal erosion. *Conclusions*: HRCT is a valuable tool in the preoperative assessment of cholesteatoma, helping in making surgical decisions. It can accurately predict the extent of disease and is helpful for detecting unapparent dangerous situations. However, it is not very accurate in detecting fallopian canal and stapes erosion.

## 1. Introduction

When discussing a topic, we usually start with its definition. When the topic is cholesteatoma, the problems already start there, and they continue in almost every aspect; it is quite difficult to name one where there are no controversies. The old definition of “skin in the wrong place” is more misleading than helpful. Cholesteatoma is a benign cystic lesion involving the temporal bone, which is derived from an abnormal growth of keratinizing squamous epithelium [1]. It consists of an outer lining of stratified squamous epithelium, an inner keratin mass, and an external peri-matrix that produces enzymes, destroying the surrounding tissues [2]. Middle ear cholesteatoma has a higher incidence in individuals younger than 50 years of age but can occur at any age [3]. Cholesteatoma in children tends to behave in a different manner than in adults; it has a more aggressive pattern of growth and recurs more frequently. 

The clinical features of cholesteatoma vary considerably, ranging from an asymptomatic disease to life-threatening complications [4]. Even if the complication rate has decreased significantly today, the danger is still there, and a warning sign of any kind is always helpful for the physician. The management of this disease is strictly surgical, continues to be a challenge for otolaryngologists, and is sometimes quite a nightmare for both patient and surgeon. 

The temporal bone has a very complicated architecture and contains delicate structures like the ossicular chain, inner ear, facial nerve, and even vital ones like the internal carotid artery [3]. Cholesteatoma may damage all of them, and it also may involve adjacent structures like the dura mater, the temporal lobe of the brain, and the sigmoid sinus, leading to both extra-cranial and intra-cranial complications as local invasion is a characteristic of the disease. It is considered to be a benign lesion, but given the complications encountered, one cannot be so sure this is the right word. 

There are also many anatomic variants of the structures in the middle ear, and the very narrow space inside the temporal bone makes discovering the lesions and tackling the complications quite challenging, so any preoperative information is highly appreciated [5]. 

HRCT of the temporal bone is one of the imaging modalities used to evaluate the extent of the disease and the lesions produced by cholesteatoma in and out of the temporal bone prior to surgery, and it is considered to be accurate as its slice thickness is usually less than 1 mm. Using special algorithms, HRCT scans offer an excellent resolution [6]. On HRCT scans, cholesteatoma appears as a soft-tissue mass in the middle ear cavity and mastoid with associated signs of surrounding bony erosion [3]. However, HRCT cannot accurately differentiate cholesteatoma from inflammatory/granulation tissue or scar tissue inside the middle ear [7]. The sinus tympani and facial recess are two hidden areas of the middle ear, and their involvement by cholesteatoma can be detected intraoperatively, especially if using an endoscope, but can also be identified preoperatively on an HRCT scan. 

In attic cholesteatoma, erosion of the scutum in the coronal view can be assessed on HRCT scans, and it is a useful sign for early diagnosis [8]. Ossicular chain erosion is a common feature in cholesteatoma, which can result in more or less significant conductive hearing loss. It can vary from small erosion of the long process of the incus and incudostapedial joint, which is the most frequent situation, to complete destruction of the entire ossicular chain. The interposition of the cholesteatoma between the tympanic membrane and stapes, no matter if it is intact or if its superstructure is eroded, can improve the sound transmission to the inner ear and be deceiving regarding the patient’s actual hearing level. Another dangerous site of erosion is the dome of the lateral semicircular canal (LSC), just above the second genu of the facial nerve canal. Speaking of the facial nerve canal, the most frequent portion that can be dehiscent or eroded in cases of cholesteatoma is the tympanic one, usually just above the oval window area [5]. These are some of the reasons why such preoperative “inside information” is useful for planning the surgical approach, and this can be best achieved using HRCT. Early detection and treatment of such dangerous situations have considerably reduced the morbidity and mortality of cholesteatoma [9]. 

The tremendous amount of information provided by the HRCT of the temporal bone has led to several studies in the literature trying to double-check the accuracy of this information by comparing it to intraoperative findings. One of the first studies to evaluate the correlation between surgical and HRCT findings in cholesteatoma was performed by Jackler in 1984 [7]. Since then, the technology behind HRCT has evolved in a spectacular manner, providing more and more details regarding the lesions in the middle ear. Most of these studies confirm a very good general correlation between HRCT and intraoperative findings, but when it comes to specific small lesions—the erosion of the stapes or a situation that can occur in normal ears as well like facial nerve canal dehiscence—this is not always the case [10,11,12]. The biggest limitation of these studies is the small number of patients—usually less than 100.

## 2. Materials and Methods

This is a prospective descriptive study conducted in the Department of Otolaryngology, Head and Neck Surgery of the Victor Babes University of Medicine and Pharmacy Timisoara, Romania, from May 2021 to April 2022. The study protocol was approved by the Ethics Committee of Victor Babes University of Medicine and Pharmacy Timisoara. The present study included 46 patients with a clinical diagnosis of chronic otitis media with cholesteatoma, in which we performed tympano-mastoid surgery, both open and closed techniques, with ossicular chain reconstruction. All patients received preoperative full clinical and audiological examination. Collected clinical data included information about tympanic cavity status (tympanic membrane, ossicles, and mucosa lesions), hearing and vestibular status, and facial nerve function. All patients underwent preoperative HRCT of the temporal bone as part of the mandatory investigations. All surgeries were performed between 3 and 8 weeks after imaging by the same surgical team under the surgical microscope. We only used the endoscope to assess the extension of the disease to sinus tympani and to check for the complete removal of the lesions. Surgical findings were recorded by the surgical team. 

The choice between the open and closed technique was made on a case-by-case basis. However, whenever possible, we went for a closed technique, or we tried to obliterate the mastoid cavity. HRCT also provided information regarding the size of the mastoid, the level of the tegmen tympani, and the position of the lateral sinus, details that are crucial when choosing the type of surgery. The extent of mastoid pneumatisation is another detail that may influence the surgical approach. If the pneumatization is good, then a closed technique can be employed easily. If there is poor pneumatisation, then an open technique is preferable with or without mastoid obliteration. However, this is not the only factor to be taken into account. The location and extension of the cholesteatoma is another crucial one, and it is very well described on an HRCT.

Interpretation of preoperative HRCT images was focused on defining the following: location and extent of cholesteatoma; bony erosions of the following structures: scutum, tegmen tympani, facial nerve (FN) canal, and inner ear (LSC, promontory); and integrity of the ossicular chain. All HRCT scans were assessed by a single radiologist with over 15 years of experience in head and neck imaging. 

The operative findings were compared to the radiological findings, assessing the usefulness of a preoperative HRCT scan in describing the status of the middle ear structures in cases of cholesteatoma with or without complications. Sensitivity, specificity, positive predictive value (PPV), negative predictive value (NPV) of HRCT scans, and Cohen’s Kappa coefficient (to evaluate their agreement with the intraoperative findings) were calculated for the following features, considered to be of interest for surgery: scutum, tegmen tympani, sigmoid sinus plate, LSC, FN canal, malleus, incus, and stapes. For statistical analysis, we used Microsoft Excel 2013 and Statistical Package for Social Sciences (SPSS) version 26 (IBM Corp., Armonk, NY, USA). The kappa coefficient in the ranges of 0–0.40, 0.41–0.75, and 0.76–1.0 indicates poor, moderate-to-good, and very good correlation, respectively. 

We did not include in the study cases with any kind of previous surgery in the same ear, cholesteatoma recurrences, and patients who were operated on later than 8 weeks after performing imaging. Written informed consent was obtained from all patients prior to data collection. 

### Scan Protocol

HRCT scans were obtained on GE Revolution EVO (General Electric, Boston, MA, USA), a 128-slice CT scanner. The patient was lying in the supine position. The topogram was made in the lateral incidence, which includes the area between the tip of the mastoid and the arcuate eminence of the temporal bone as the lower and upper scanning point, respectively; scanning was not performed with an inclined gantry. 

Axial projections were obtained by serial 1 mm sections of the temporal bone with the plane along the line joining the infra-orbital rim and external auditory meatus, perpendicular to the table with 20 × 0.625 collimation. The images were reconstructed using the high-resolution bone algorithm in the axial plane with 0.625 mm section thickness and a field of view (FOV) of 100–110 mm, with a matrix size of 512 × 512. The effective amperage in the tube was modulated according to the patient’s age: between 130 mAs (for a newborn) and 400 mAs (for an adult). The voltage in the tube was between 110 and 140 kV, usually 120 kV. 

The acquisition was in the spiral mode, which ensures a better reformation in the coronal and sagittal plane. The reconstruction in the axial plane is the basic one and includes sections parallel to the lateral semicircular canal; simultaneously with the reconstruction in the three planes, a set of images in the axial plane with a thickness of 2 mm was also obtained, which helps to visualize the tissue structures within the cholesteatoma as a whole.

The administration of the contrast substance was not always indicated in cholesteatoma involving the middle ear, but it becomes mandatory in situations where there is a suspicion of evolution towards complication: abscesses, coalescent otomastoiditis, and sigmoid sinus thrombosis.

## 3. Results

Chronic otitis media with cholesteatoma represents 41% of the chronic otitis media cases operated in our department in 2022. There were 46 patients enrolled in our study, including 6 children ages 8 to 15 years old. The oldest patient was 73 years old. The mean age of our patients was 37.3 ± 10.8 years. The largest number of cases, 19 (41%), were in the 31–40-year-old group, and among them 11 (57%) were male and 8 (43%) were female. There was generally a slight male preponderance in our study population—24 (52%) male and 22 (48%) female patients—with a male/female ratio of 1.09:1. Extensive cholesteatoma was the most commonly encountered type, detected in 21 patients (46%), and followed by pars flaccida cholesteatoma encountered in 16 patients (35%) and pars tensa cholesteatoma in 9 patients (19%). 

The erosion of the scutum was the most common lesion, but only by a narrow margin, and was found in 32 patients (70%). The other destructive lesions were eroded incus, mainly the long process, in 31 patients (67%), eroded malleus in 21 patients (46%), eroded stapes in 14 patients (30%), significantly thinned/eroded tegmen tympani in 10 patients (22%), dehiscent/eroded facial nerve canal in 8 cases (17%), lateral semicircular canal fistula in 4 cases (9%), and eroded sigmoid sinus plate in 3 cases (7%). The patients enrolled in this study presented the following complications: two patients with facial nerve palsy (grade III and IV House–Brackmann, respectively) and four patients with vertigo. All the patients with vertigo were correctly assessed from an imaging point of view. There was an extra case described as having a lateral semicircular canal fistula on HRCT scans, which was not confirmed during surgery. When analyzing alterations of tegmen tympani, we considered both erosion and thinning together as their clinical and surgical significance is comparable. 

Table 1 shows the sensitivity, specificity, positive predictive and negative predictive values, and correlation levels for the considered HRCT findings.

The greatest correlation between imaging and surgery was in the detection of the tegmen tympani erosion/thinning (kappa = 0.97). We also obtained a very good imaging-surgical correlation in the detection of sigmoid sinus plate dehiscence (kappa = 0.87), malleus erosion (kappa = 0.82), and scutum erosion (kappa = 0.76). We obtained moderate-to-good results in detecting lateral semicircular canal dehiscence (kappa = 0.68), incus erosion (kappa = 0.62), stapes erosion (kappa = 0.44), and facial nerve canal dehiscence (kappa = 0.42). There was no poor imaging-surgical correlation for any of the investigated lesions in our study. The poorest correlation was in facial nerve canal dehiscence/erosion (kappa = 0.42) and stapes erosion (kappa = 0.44). The best correlation was in cases with extensive and severe lesions, as it was expected preoperatively. 

## 4. Discussion

The ability of HRCT to detect cholesteatoma and its complications prior to surgery is well known. It is especially helpful in detecting minimal erosion of the ossicular chain and other dangerous areas like the tegmen tympani, lateral semicircular canal, and fallopian canal [10]. Such information is very useful when discussing with the patient and getting informed consent for surgery. It is still worth remembering that HRCT cannot accurately differentiate cholesteatoma from granulation tissue and cholesterol granuloma, which are also encountered in chronic suppurative otitis media. All our cases had a clinical diagnosis of cholesteatoma prior to imaging, so this was not our main concern, but cholesteatoma was confirmed, and its extension was accurately described on imaging.

Age—In our study, we found that the majority of patients were aged between 31 and 40 years of age, involving 19 (41%) cases. In other studies, a much younger age prevalence of less than 30 years of age was observed [11,12].

Gender prevalence—In the present study, the majority of patients were male (52%), but only by a thin margin, with a male-to-female ratio of 1.09: 1. Kemppainen et al. also reported that cholesteatoma was more frequent in men under the age of 50 years [13]. Other studies reported a much higher male predominance, with a male/female ratio of 1.39:1 [14].

Location and extent of cholesteatoma—In our study, extensive cholesteatoma was the most commonly encountered type, detected in 46% of patients, followed by pars flaccida cholesteatoma in 35% of patients and pars tensa cholesteatoma present in 19% of patients. The same prevalence was observed in the study performed by Gomma et al., where extensive cholesteatoma was detected in 35.7% of patients. Also commonly detected was the pars flaccida type [12]. In other studies, the most frequently encountered type was pars flaccida cholesteatoma: a study on 30 cholesteatoma cases showed lesions in the epitympanum in 73.3% of patients, followed by mesotympanum [15], and in another one pars flaccida cholesteatoma was described in 50.7% of cases, followed by extensive cholesteatoma in 30.1% of patients [16]. Pars tensa cholesteatoma was the least encountered type in most of the studies.

Accurate assessment of the extension of the lesions may provide crucial information regarding the most suitable surgical approach considering the given anatomy of the middle ear and mastoid. It is notorious for the controversy among the surgeons supporting either the open or the closed technique in the treatment of cholesteatoma. However, the closed technique might be extremely difficult in some temporal bones, and this can be foreseen in imaging. There are also situations in which an open technique is not required because of the limited extension of cholesteatoma [17].

From a surgical point of view, the accuracy in detecting lesions of the delicate structures of the middle ear presents great importance, and this is why several aspects regarding this topic deserve some comments.

Scutum erosion—It is considered to be an early sign of pars flaccida cholesteatoma, but it does not appear in all cases. In our study, it was detected in 32 cases (70%) with a sensitivity of 87.5% and a specificity of 92.8%. The correlation was still very good but borderline (kappa = 0.76). In another study, the scutum was eroded in 55.5% of the cholesteatoma cases [16]. Rai et al. reported scutum erosion in 65% of the patients [18]. The PPV was very good in our study (96.5%). In other studies, scutum erosion was accurately predicted in 91% of cases [19,20]. However, Sunitha reported no imaging-surgical correlation for scutum erosion [21]. Suat Keskin believes that differences between imaging and intraoperative findings regarding scutum erosion may be due to inappropriate angles of the coronal sections [22]. From a surgical point of view, the erosion of the scutum may influence the approach to the ossicular chain, especially to the incus and malleus.

Sinus plate erosion—In our study, erosion of the sigmoid sinus plate was described on HRCT in four cases, but intraoperatively, only three cases had sinus plate erosion, so only 75% of cases were accurately detected by HRCT with relatively lower sensitivity when compared to other studies. In a study by Dutta et al., HRCT sensitivity was 100%, and specificity was also 100% for sinus plate erosion [11]. C Shah reported the sensitivity and specificity of HRCT in detecting sigmoid erosion to be 91.7% and 95.25%, respectively [17]. Kanotra et al. reported the sensitivity, specificity, and positive and negative predictive value to be 100% [23]. Sinus plate erosion is quite a rare occurrence, and this can explain why there are such differences between studies. Knowing that the sinus plate is eroded requires special attention and careful exploration of the region, even if there are no clinical signs of sigmoid sinus pathology. It can also alert the surgeon and prevent the inadvertent opening of the sinus wall with massive bleeding and prolonged surgical time.

Tegmen tympani erosion/thinning—We evaluated erosion and significant thinning of the tegmen tympani together, but not all studies did so. This is probably one reason why we obtained such good specificity and sensitivity compared to others. HRCT finding shows eroded/thinned tegmen tympani in 13 cases, but intraoperatively, it was present in 9 cases out of the total of 46 (19%) with a sensitivity of 90% and specificity of 88.8%. Another study identified tegmen tympani erosion in 38% of the cases [16]. In a study performed by Jamal et al., tegmen tympani erosion was seen in 30% of the patients [24]. Kanotra et al. reported a sensitivity of 100% in detecting tegmen tympani erosion by HRCT, which was higher than the current study [23]. A high specificity rate of 95% was reported by Gerami et al. [25]. A poor sensitivity rate of HRCT in detecting tegmen tympani erosion was reported by Jackler et al. [9] and O’Reilly et al. [26], while a moderate sensitivity rate was described by Vlastarakos et al. [27] and Chee and Tan [10]. From a surgical point of view, it is invaluable information for the dissection of the cholesteatoma matrix away from that area, even in the absence of dura-related complications. The poor imaging-surgical correlation for tegmen tympani erosion is probably due to the partial volume of both the tympanic cavity and cerebral soft tissue [22].

Lateral semicircular canal erosion—This structure may be eroded by cholesteatoma, especially in its dome region on the medial wall of the epitympanum, close to the second genu of the facial nerve [3]. In our study, we found four cases (9%) of an LSC fistula. Other studies noted an LSC fistula in just 4.7% of the cases and even lower (4% of the patients) [16,28]. In our study, HRCT scans assessed the LSC fistula cases with a sensitivity of 75% and specificity of 97.6%. In a study performed by Gaurano et al. on 64 patients, there were four cases (6.3%) that had labyrinthine fistula found on HRCT, but only three (4.7%) were confirmed intraoperatively [19]. Usually, HRCT accurately predicts lateral semicircular canal fistula with excellent sensitivity and good specificity. Mafee et al., Chee et al., and Rocher et al. reported HRCT to be 100% sensitive in detecting lateral semicircular canal fistula [3,10,20]. Knowing in advance about the suspicion and location of the fistula is very important for the surgeon as dissection of the matrix of the cholesteatoma in that area has to be performed very carefully, avoiding unnecessary manipulation and suction. The closure of the fistula is of utmost importance for inner ear function.

FN Canal dehiscence/erosion—Fallopian canal dehiscence/erosion is a relatively common finding in cholesteatoma affecting the middle ear, usually occurring in the tympanic portion of the facial nerve canal [8,29]. We found facial nerve canal dehiscence/erosion in eight patients (17%). In another study, facial canal dehiscence was observed in 34.9% of the cholesteatoma cases [16]. The study by Jamal et al. reported facial canal dehiscence in 30% of the patients [24]. The sensitivity and specificity obtained in our study were 50% and 92.1%, respectively, with a moderate-to-poor correlation between imaging and surgery (kappa = 0.42). Other studies presented similar observations with a sensitivity ranging from 33.3% to 83% and specificity between 60% and 97%. It is well known that the Fallopian canal can be so thin as to appear dehiscent on a CT scan [30]. It is good to remember that the fallopian canal may be dehiscent even in a healthy ear. The detection of the facial nerve dehiscence/erosion on imaging should influence the decision and timing of surgery, being a good reason to operate earlier and alert the surgeon when working in that area. One should always consider the proximity of the stapes and facial nerve, especially when dissecting the cholesteatoma matrix off these structures.

Ossicular chain—It is usually involved by cholesteatoma of the middle ear, even if not always eroded or discontinued. This situation presents great importance for the surgeon as he/she has to drill and dissect a lot around the ossicular chain. Touching the ossicular chain during drilling might generate severe SNHL and should be avoided at any cost. We found ossicular chain erosion in 37 out of 46 patients (80%). Incus was the most eroded ossicle, found in 67% of the patients in our study. In another study, incus was found to be eroded in 60.3% of the cases, followed by malleus in 58.7% and stapes in 47.6% of the patients [16]. Manik et al. also showed incus to be the most commonly affected ossicle in 70% of the patients, followed by malleus in 42% and stapes in 34% of the patients [31]. In our study, the most accurate information was regarding the erosion of the malleus, the head of the malleus being the most affected site, with a sensitivity of 85.7% and a specificity of 96% (kappa = 0.82). It was followed by erosion of the incus, with a sensitivity of 80.6 and specificity of 86.6% (kappa = 0.62). Other authors observed comparable sensitivity of 87% and 85%, respectively [11,18]. The stapes is the least eroded ossicle in most of the studies. However, the poorest imaging-surgical correlation was regarding stapes erosion. In our study, sensitivity was 57.1%, and specificity was 84.3% (kappa = 0.44), which is borderline between moderate and poor correlation. Other authors also reported poor radiologic-surgical correlation in stapes erosion and stated that the small size of the bone may be the cause of poor detection on HRCT scans [32,33]. However, if an ossicle is involved by the cholesteatoma, it has to be removed, no matter if it is eroded or not. The only exception is the stapes footplate, in which case the cholesteatoma matrix should be carefully dissected off the structure. Anything else can and should be removed, so preoperative information regarding the erosion of the malleus, incus, and even stapes superstructure is not essential for the ossicular chain reconstruction. The only unknown factor is which type of prosthesis should be used for the reconstruction of the sound transmission mechanism, and this depends on the status of the stapes superstructure—present and functional or absent/eroded. Either way, the stapes footplate needs to be mobile. However, you can always use a total ossicular replacement prosthesis (TORP) even if the stapes superstructure is intact by bypassing it.

HRCT is still considered the best available imaging method to describe the cholesteatoma of the middle ear, benefiting both the patient and the surgeon. Early diagnosis of cholesteatoma is also crucial, and there are important findings that can alert the physician, such as erosion of the scutum. MRI is another useful tool in cholesteatoma patient evaluation. Its main role is especially in the follow-up of patients with previous surgery since recurrence or residual cholesteatoma can be well detected, avoiding the risk of unnecessary revision surgery (second look operation). Preoperatively, MRI is also very useful in detecting intracranial complications. In both these circumstances, MRI is more helpful than HRCT.

This study demonstrated a good-to-excellent correlation between temporal bone HRCT findings and intraoperative lesions, particularly in tegmen tympani erosion, sigmoid plate dehiscence, malleus erosion, and scutum erosion. Our findings contrast with other reports, which showed an excellent radio-surgical correlation for the stapes (kappa = 0.94) and semicircular canals (kappa = 0.80), but poorer for the tegmen tympani erosion/thinning (kappa = 0.65) and facial nerve canal (kappa = 0.3) [10]. Rocher and colleagues revealed a good-to-excellent correlation for the scutum erosion and the lateral semicircular canal dehiscence (kappa > 0.75) and a good correlation for tegmen tympani erosion (k = 0.6) but a poor correlation for the facial nerve canal dehiscence (kappa < 0.4) [20].

The very good correlation between the findings on HRCT scans and intra-operative lesions may determine a higher degree of suspicion for unexpected problems during surgery and may improve the success rate of cholesteatoma surgery. The limitations and errors in interpretation may be improved by newer imaging technology.

The main limitation of this study consists of the small number of patients included and the small number of dangerous lesions encountered: eroded tegmen tympany in nine cases (19%), lateral semicircular canal fistula in four cases (9%), and eroded sigmoid sinus plate in three cases (7%).

## 5. Conclusions

The results of the present study indicate that HRCT is a most valuable preoperative imaging modality to evaluate cholesteatoma of the middle ear, playing an important role and guiding surgical management. HRCT scans can accurately predict the extent of the disease and are helpful in detecting erosion of the tegmen tympani, lateral semicircular canal fistulas, sigmoid sinus plates, and ossicular chain erosion with considerably high sensitivity and specificity. Accurate information on cholesteatoma lesions that are offered to the surgeon by means of HRCT allows for more limited procedures to be performed when eradicating the disease while preserving the function of the middle and inner ear and can alert the surgeon over dangerous but “silent” complications making him operate early. However, this imaging technique is not yet able to accurately distinguish between cholesteatoma and granulation tissue and cannot accurately detect facial nerve dehiscence/erosion and stapes erosion. Considering the characteristics—more aggressive and more prone to recurrences—cholesteatoma in children should deserve a separate study regarding the correlation between HRCT and intraoperative findings but on a more significant number of cases.

## Figures and Tables

**Table 1:** Accuracy of preoperative HRCT in detecting cholesteatoma lesions.

HRCT Findings	Sensitivity	Specificity	PPV	NPV	Correlation
Tegmen tympani erosion/thinning	90.0	88.8	69.2	96.9	0.97
Sigmoid plate erosion	100	97.6	75.0	100	0.87
Malleus erosion	85.7	96.0	94.7	88.8	0.82
Scutum erosion	87.5	92.8	96.5	76.4	0.76
LSC erosion	75.0	97.6	75.0	97.6	0.68
Incus erosion	80.6	86.6	92.5	68.4	0.62
Stapes erosion	57.1	84.3	61.5	81.8	0.44
Facial canal dehiscence/erosion	50.0	92.1	57.0	89.7	0.42

## Data Availability

Data supporting reported results can be found in the archives of the Department of Otolaryngology and Department of Radiology and Medical Imaging of the Victor Babes University of Medicine and Pharmacy Timisoara.
